# Zileuton protects pancreatic stellate cells from oxidative stress and ferroptosis by modulating the Nrf2 pathway and mitochondrial activity

**DOI:** 10.1007/s13105-026-01224-9

**Published:** 2026-09-16

**Authors:** Matías Estarás, Cándido Ortiz-Placín, Salomé Martínez-Morcillo, Remigio Martínez, Miguel Fernández-Bermejo, Pablo Solís-Muñoz, José María Mateos, Juan Iovanna, Patricia Santofimia-Castaño, Antonio Gonzalez

**Affiliations:** 1 Grupo de Investigación Biología y Comunicación Celular. Departamento de Fisiología, Instituto de Biomarcadores de Patologías Moleculares, Facultad de Veterinaria, Universidad de Extremadura, Avenida de la Universidad s/n, Cáceres, Spain; 2 Unidad de Toxicología, Facultad de Veterinaria, Universidad de Extremadura, Avenida de la Universidad s/n, Cáceres, Spain; 3 Departamento de Sanidad Animal, Facultad de Veterinaria, Universidad de Extremadura, Cáceres, Spain; 4 Unidad de Gastroenterología, Complejo Hospitalario de Cáceres, Cáceres, Spain; 5 Centre de Recherche en Cancérologie de Marseille (CRCM), INSERM U1068, CNRS UMR 7258, Aix-Marseille Université and Institut Paoli- Calmettes, Parc Scientifique et Technologique de Luminy, 163 Avenue de Luminy, Marseille, 13288 France; 6 Institute of Molecular Pathology Biomarkers, Department of Physiology, University of Extremadura, Avenida de las Ciencias s/n, E- 10003 Caceres, Spain

**Keywords:** Zileuton, Cell viability, Glutathione, Ferroptosis, Nuclear factor erythroid 2-related factor, Pancreatic stellate cells, Antioxidant enzymes

## Abstract

Lipoxygenases exhibit a leading role in cancer development and growth. Additionally, pancreatic stellate cells (PSCs) are pivotal in the formation of fibrotic tissue in pancreatic tumors. In this work, we have studied the effects of the 5-lipoxygenase (5-LOX) inhibitor, zileuton, on rat PSCs. Cell viability was analyzed by employing crystal violet. Reactive oxygen species (ROS) generation, reduced (GSH) and oxidized (GSSG) levels of glutathione and protein and lipid oxidation were analyzed by fluorescence and colorimetric methods respectively. The phosphorylation of nuclear factor erythroid 2-related factor (Nrf2) and the expression of superoxide dismutase (SOD) was studied by western blotting. The effect of zileuton on erastin-induced ferroptosis was assessed by colorimetric methods. Mitochondrial metabolic activity was studied employing Alamar Blue^®^. Mitochondrial membrane potential was monitored by fluorescence techniques. The results show that the 5-LOX inhibitor protected PSCs against oxidation evoked by hydrogen peroxide, which induced an increase in CM-H_2_DCFDA-derived fluorescence, a decrease in the reduced/oxidized glutathione ratio and increases in the detection of lipid and protein oxidation. In the presence of zileuton, increases in the phosphorylation of nuclear factor erythroid 2-related factor and in the expression of the antioxidant enzymes catalase, glutamate-cysteine ligase,,heme oxygenase-1, NAD(P)H quinone oxidoreductase 1, and superoxide dismutases 1 and 2 were observed. Additionally, zileuton diminished erastin-evoked ferroptosis. Finally, a drop in the reduction of resazurin by mitochondria and a slowing of the Krebs cycle flow rate were observed, whereas no detectable changes in mitochondrial membrane potential were noted in the presence of zileuton. We conclude that the inhibition of 5-LOX by zileuton places cells in a state of antioxidant readiness coupled with a protected metabolic deceleration.

**Supplementary Information:**

The online version contains supplementary material available at https://doi.org/10.1007/s13105-026-01224-9.

## Introduction

Pancreatic stellate cells (PSCs) constitute a significant component of the pancreatic stroma. Despite they represent a small cell population of the organ under a physiological state, emerging evidence strongly suggests a pivotal role for these cells in the pathogenesis of various pancreatic diseases [1]. This observation is based on the principle that under physiological conditions, PSCs reside in a non-activated, quiescent, state. However, upon injury or insult, these cells undergo transitioning to a so-called activated state, which is implicated in the pathogenesis of various pancreatic diseases. In this context, activated PSCs are critical contributors to progressive fibrosis and the accumulation of extracellular matrix, characteristic of chronic pancreatitis and pancreatic cancer [2]. Moreover, PSCs contribute actively the extension of the fibrotic reaction by the release of signaling molecules that act on neighboring cells to modulate their proliferation and tissue growth within cancer [3]. In all this interplay, the generation of reactive oxygen species (ROS), together with the concomitant change in the oxidative state, appears as a driving force that determines PSCs´ fate [4, 5]. Therefore, elucidating the mechanisms governing the growth and proliferation of PSCs is of paramount importance for comprehending the pathogenesis of pancreatic diseases.

Arachidonic acid (AA) is a major constituent of the cell membrane phospholipids. Upon release by the action of the enzyme phospholipase A2 (PLA2), this lipid can be transformed into several bioactive compounds by a family of enzymes. The components released after its metabolic transformation play critical roles in cell physiology and, moreover, are involved in the development of pancreatic disease [6]. Apart from the other enzymes involved, 5-Lipoxygenase (5-LOX) is a major AA-metabolizing enzyme that has been implicated in the generation of eicosanoids, which contribute to pancreatic cancer cell proliferation [7]. In this line, down-regulation of 5-LOX expression and its downstream leukotriene B4 production inhibited cell growth and induced death in human pancreatic cell lines [8]. Zileuton is a proven 5-LOX inhibitor [9]. It has been shown that this compound exerts promising anticancer effects in several tissues [10–12]. Nonetheless, despite its prominent role in pancreatitis and in the development and growth of pancreatic tumors, studies on the effect of 5-LOX inhibition on the physiology or pathophysiology of PSCs are currently lacking.

In the present study, we aimed at identifying the actions of zileuton, a 5-LOX inhibitor, on rat PSCs. Understanding the intracellular pathways crucial for the function of this cell population may be essential for designing therapeutic targets and diagnostic biomarkers for pathologies associated with the development of pancreatic fibrosis.

## Materials and methods

### Cells and chemicals

Cultures of pancreatic stellate cells were prepared from pancreatic tissues that were obtained from newborn *Wistar* rats (5 days). Animals were purchased from the animal house of the University of Extremadura (Caceres, Spain). Handling and experimental protocols were approved by the Ethical Committee for Animal Research of the University of Extremadura (reference 44/2024). AlamarBlue^®^ was purchased from Bio-Rad (C-VIRAL, Madrid, Spain). Collagenase CLSPA was obtained from Worthington Biochemical Corporation (Labclinics, Madrid, Spain). Carbonyl cyanide-4-(trifluoromethoxy)phenylhydrazone (FCCP), Cell Lysis Reagent for cell lysis and protein solubilization, crystal violet, hydrogen peroxide (H_2_O_2_), oxidized glutathione, *o*-Phthalaldehyde, protease inhibitor cocktail (Complete, EDTA-free), reduced glutathione, thapsigargin, Tween^®^-20 and zileuton were obtained from Sigma-Aldrich (Merck Life Science S.L.U.; Madrid, Spain). 5-(and-6)-chloromethyl-2’,7’-dichlorodihydrofluorescein diacetate acetyl ester (CM-H_2_DCFDA), fetal bovine serum (FBS), Hank’s balanced salts (HBSS), horse serum and medium 199 were purchased from Invitrogen (Fisher Scientific Inc., Madrid, Spain). Fluorescent probes for mitochondrial monitorization Hoechst 33,342, MitoTracker™ Green FM and tetramethylrhodamine methyl ester perchlorate (TMRM) were purchased from Invitrogen (Fisher Scientific Inc., Madrid, Spain). Polystyrene plates for cell culture were purchased from Thermo Fisher Sci. (Madrid, Spain). Penicillin/Streptomycin was obtained from BioWhittaker (Lonza, Basel, Switzerland). Bradford reagent, Tris/glycine/SDS buffer (10×) and Tris/glycine buffer (10×) were from Bio-Rad (Madrid, Spain). SignalFire™ ECL Reagent was obtained from Cell Signaling Technology (C-Viral, Madrid, Spain).

Antibodies against phosphorylated Nrf-2 (Ser40), against SOD1 and primers for RT-qPCR were purchased from Thermo Fisher Sci. (Madrid, Spain). Antibody against SOD-2 was obtained from Santa Cruz Biotechnologies Inc. (Quimigen S.L., Madrid, Spain). Antibody against Nrf2 was purchased from Abcam (VWR International Eurolab S.L.U., Barcelona, Spain). Anti-actin antibody was purchased from Sigma-Aldrich (Merck Life Science S.L.U.; Madrid, Spain). Secondary antibodies goat anti-rabbit IgG HRP-conjugate and goat anti-mouse HRP-conjugate were purchased Thermo Fisher Sci. (Madrid, Spain). All other analytical grade chemicals used were obtained from Sigma-Aldrich (Merck Life Science S.L.U.; Madrid, Spain).

### Culture of rat pancreatic stellate cells

PSCs were prepared and cultured using previously described methods [13]. Cells were seeded on polystyrene plates for cell culture. Culture medium comprised of medium 199 supplemented with 4% horse serum, 10% FBS, a mixture of antibiotics (0.1 mg/mL streptomycin, 100 IU penicillin) and 1 mM NaHCO_3_). The cells were grown in a humidified incubator with controlled temperature (37 °C) and CO_2_ (5%). Confluence (90–95%) was reached after eight-ten days of culture. Different cell preparations were used in the studies.

### Determination of reactive oxygen species generation

Cells were detached and loaded with the fluorescent probe CM-H_2_DCFDA (10 µM) as described previously [14]. Then, different aliquots of dye-loaded cells were incubated with stimuli for 1 h. Red-ox state of cells was monitored by measuring cellular fluorescence at 530 nm/590 nm (excitation/emission) for CM-H_2_DCFDA. A plate reader CLARIOstar Plus was used (BMG Labtech., C-Viral, Madrid, Spain). The experiments were conducted employing batches of cells obtained from different preparations. Results show the mean increase of fluorescence expressed in percentage ± SEM (n) with respect to non-stimulated cells (n is the number of independent experiments).

### Determination of glutathione levels

The changes in the levels of reduced (GSH) and oxidized (GSSG) glutathione were determined using methods described previously [15]. Cells were incubated for 4 h with stimuli. A plate reader CLARIOstar Plus was used (BMG Labtech., C-Viral, Madrid, Spain) was employed to detect GSH or GSSG at 350 nm/420 nm (excitation/emission) respectively. Standard curves of GSH and GSSG were used for quantification. Normalization was carried out based on the total protein concentration in each sample and using a standard curve prepared with bovine serum albumin [16]. The experiments were carried out employing batches of cells obtained from different preparations. Data are shown as the mean increase in GSH/GSSG ratio expressed in percentage ± SEM (n) with respect to non-stimulated cells (n is the number of independent experiments).

### Determination of protein carbonyls (Allysine)

After treatment, cells were washed with standard phosphate saline solution and were lysed for analysis. Protein carbonyls were determined following methods used previously [5]. Results are expressed as nanomoles (nmol) of allysine per mg of protein content ± SEM (n) with respect to non-treated cells (n is the number of independent experiments).

### Analysis of thiobarbituric-reactive substances

After treatment, cells were lysed for analysis. Malondialdehyde (MDA) and other thiobarbituric acid reactive substances (TBARS) were measured in 200 µL samples of each treatment using previously described methods [5]. Results are expressed as nanograms (ng) of TBARS per mg or protein ± SEM (n) with respect to non-treated cells (n is the number of independent experiments).

### Study of oxidation-reduction (REDOX) with AlamarBlue^®^

PSCs were seeded in 96 well plates at a density of 5000 cells/well. AlamarBlue^®^ reagent was added to the culture medium (10% v/v) prior to addition of drugs. At the desired time, fluorescence was monitored at 530 nm excitation and 590 nm emission wavelengths employing a plate reader (CLARIOstar Plus. BMG Labtech., C-Viral, Madrid, Spain), according to the manufacturer´s directions. Data show the mean reduction of AlamarBlue^®^ expressed in percentage ± S.E.M. (n) with respect to the values noted in non-treated cells (incubated in the absence of stimulus), where n is the number of independent experiments.

### Confocal microscopy

This procedure was carried out following previously described methods (Estaras et al. Biochem Pharmacol., 2022) [17]. Briefly, the cells were grown on glass cover slips. For monitorization of mitochondrial distribution, cells were incubated with MitoTracker™ Green FM (100 nM; room temperature 23–25 °C, 30 min.), tetramethylrhodamine methyl ester (TMRM; 50 nM, 37 °C; 30 min.) and Hoechst 33,342 (5 µg/mL., room temperature 23–25 °C, 5 min.). Monitorization of fluorescence was performed employing a confocal laser system, Zeiss LSM 980, connected to an AxioObserver7 microscope (Carl Zeiss Iberia S.L., SSC Madrid, Spain). Cells were observed with a 40 × oil immersion objective. Fluorescence images of 2512 × 2512 pixels were recorded. The laser lines used were 488 nm for MitoTracker™ Green FM, 561 nm for TMRM and 405 nm for Hoechst.

Separate preparations of cells were loaded with TMRM to monitor changes in mitochondrial membrane potential (Ψm). Small round fluorescent areas (ROIs), consistent with mitochondria, were selected and fluorescence was monitored employing dedicated software (Zen 3.4 pro, Carl Zeiss Iberia S.L., SSC Madrid, Spain). Fluorescent areas (ROIs) from four to six different cells were randomly chosen within aleatory fields in 3 different preparations. Fluorescence images were taken every 5 s. Data are expressed as absolute values of fluorescence normalized to basal (prestimulation) level.

### Quantification of mitochondrial membrane potential

Quantification of changes in mitochondrial membrane potential (Ψm) were recorded using TMRM-derived fluorescence, following previously described methods [18]. In brief, PSCs growing on 24 mm diameter glass coverslips, were incubated during 30 min in the presence of 50 nm TMRM at 37 °C. Afterwards, coverslips with dye-loaded cells were mounted into an experimental perfusion chamber, which was placed on the stage of an epifluorescence inverted microscope (Axio Observer 3/5/7 KMAT; Carl Zeiss Iberia S.L., SSC Madrid, Spain). A Fluor 40x/1,3 oil-immersion objective was employed (Carl Zeiss Iberia S.L., SSC Madrid, Spain). The microscope was equipped with led light and filters allowing excitation of TMRM at 543 nm (Carl Zeiss Iberia S.L., SSC Madrid, Spain). Images of small round fluorescent areas (ROIs), consistent with mitochondria, were captured employing a Flash 4.0 V2 digital CMOS camera (Hamamatsu Photonics-France, Barcelona, Spain). Software ZEN 3.4 pro (Carl Zeiss Iberia S.L., SSC Madrid, Spain) was used to control the image acquisition system.

Images were analyzed with image-J software (http://imagej.nih.gov/ij/) to calculate changes in fluorescence. Mitochondrial areas were randomly chosen within the cells present in the field. The mean intensity of fluorescence was calculated after subtraction of background fluorescence. Three different cellular preparations were used. Results are expressed as the mean ± SEM (n) of fluorescence values expressed as % vs. fluorescence noted at the beginning of the experiment.

### Western blotting analysis

Western blotting was performed using previously described methods [19]. Cells were incubated in the presence of different stimuli for 1 h and lysed. Bradford´s method was used for quantification of the protein content of lysates [16]. Protein lysates (15 µg/lane) of each sample were separated by SDS-PAGE, using 10% polyacrylamide gels, and were transferred to nitrocellulose membranes. Specific primary and the correspondingIgG-HRP conjugated secondary antibody were used for detection of proteins. Density of the bands was detected using a Syngene Gbox Chemi Gel documentation system (C-Viral, Madrid, Spain). Quantification of the intensity of the bands was performed using the software Image J (http://imagej.nih.gov/ij/). The experiments were carried out employing batches of cells obtained from different preparations. Values are expressed as the mean ± SEM (n) of normalized values expressed as % vs. non-treated cells.

### Quantitative reverse transcription-polymerase chain reaction (RT-qPCR) analysis

RT-qPCR was used to detect the expression of antioxidant enzymes. Cells were subjected to treatments (4 h incubation with stimuli) and were then lysed. Lysates were thereafter used for total RNA purification and analysis of protein expression as described previously [20]. Determinations were carried out in the Research Support Services of the University of Extremadura (SAIUEx). Total RNA samples were purified using a commercially available kit (Sigma, Madrid, Spain). Reverse transcription was performed for 30 min at 48 °C, and PCR conditions were 10 min at 95 °C followed by 40 cycles of 15 s at 95 °C plus 1 min at 55 °C using the primers listed in Table 1. The mRNA abundance of each transcript was normalized to the Gapdh mRNA abundance obtained in the same sample. The relative mRNA levels were calculated using the ΔΔCt method and were expressed as the fold change between sample and calibrator. The experiments were carried out employing batches of cells obtained from different preparations.

**Table 1:** List of primers used in the study

Primer	Forward	Reverse
*Cat*	5′-ACTTTGAGGTCACCCACGAT-3′	5′-AACGGCAATAGGGGTCCTCTT-3′
*Gapdh*	5′-GGGTGTGAACCACGAGAAAT-3′	5′-CCTTCCACGATGCCAAAGTT-3′
*Gclc*	5′-GGCACAAGGACGTGCTCAAGT-3′	5′-TGCAGAGTTTCAAGAACATCG-3′
*Ho-1*	5′-AGCACAGGGTGACAGAAGAG-3′	5′-GAGGGACTCTGGTCTTTGTG-3′
*Nqo-1*	5′-GGGGACATGAACGTCATTCTCT-3′	5′-AAGACCTGGAAGCCACAGAAGC-3′

RT-qPCR was performed for 30 min at 48 °C. PCR conditions were 10 min at 95 °C followed by 40 cycles of 15 s at 95 °C plus 1 min at 55 °C using the primers listed above (purchased from Thermo Fisher; Madrid, Spain). The abundance of *Gapdh* mRNA in each sample was used for normalization (*n* = 3 independent experiments)

### Cellular viability assay

Cell density count was used to determine cellular viability. Determination of cell number in the cultures was carried out using crystal violet test following previously described methods [5]. Briefly, after treatment of cells with stimuli, cells were washed with cold standard PBS and fixed with 4% paraformaldehyde (15 min. at room temperature, 23–25 °C). Next, the cells were stained by incubation in the presence of 0.1% crystal violet (20 min. at room temperature, 23–25 °C). This incubation was followed by washing with distilled water. Thereafter, water was removed, the wells were allowed to air dry and then 10% acetic acid was added to each well of the plate. Finally, the absorbance of each sample was measured at 590 nm employing a plate reader (CLARIOstar Plus, BMG Labtech., C-Viral, Madrid, Spain). The viability of cells subjected to stimuli was compared with that of control cells (incubated in the absence of stimulus). The experiments were carried out employing batches of cells obtained from different preparations. Data are shown as the mean change of absorbance expressed in percentage ± SEM (n) with respect to non-treated cells (n is the number of independent experiments).

### Determination of Krebs cycle metabolites

PSCs were subjected to treatments (1 h incubation with stimuli) and Krebs cycle metabolites were determined. Analysis of samples was carried out in the Analysis and innovation service for animal-based products of the University of Extremadura (SiPA-SAIUEX) using ultra-high performance liquid chromatography coupled to triple quadrupole mass spectrometry, UHPLC-MS (QqQ) Agilent 6470, operating in negative electrospray ionization and Multiple Reaction Monitoring (MRM) mode with specific transitions for each metabolite. Chromatographic separation was performed using a hydrophilic interaction column, with a mobile phase consisting of 5 mM ammonium acetate in water and methanol. Quantification was carried out via external calibration using certified reference standards of the target analytes, utilizing MassHunter software. Each sample was analyzed in duplicate.

### Statistical analysis

Statistical analysis of data was performed by one-way analysis of variance (ANOVA) followed by Tukey *post hoc* test, and only *P* values < 0.05 were considered statistically significant. For individual comparisons and statistics between individual treatments we employed the Student’s *t* test, and only *P* values < 0.05 were considered statistically significant.

## Results

### Effect of zileuton on cellular viability

**Fig. 1:**
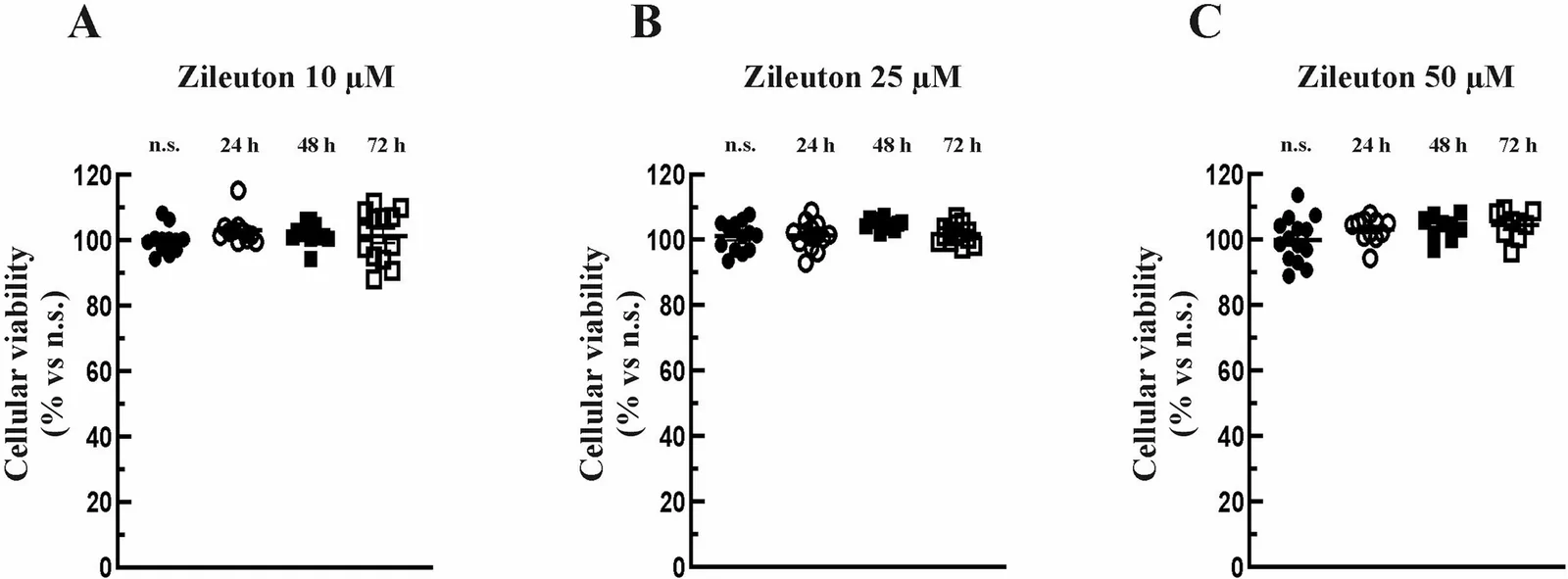
Effect of zileuton on the viability of PSCs. Different batches of cells were incubated for 24 h, 48 h and 72 h in the absence (non-treated) or in the presence of zileuton 10 µM (A), 25 µM (B) or 50 µM (C). Viability of cells subjected to treatments with the 5-LOX inhibitor was compared with that of cells incubated in its absence (non-treated cells). The graphs show the mean increase in cellular viability expressed in percentage ± S.E.M. with respect to cells incubated in the absence of drugs (n.s., non-treated cells). The experiments shown are representative of three different preparations

In a first instance, we studied the effect of various concentrations of zileuton on the viability of PSCs. For this purpose, cells were incubated in the presence of 10 µM, 25 µM or 50 µM zileuton for 24 h, 48 h and 72 h. In the presence of the 5-LOX inhibitor we did not observe significant changes in cell viability (Fig. 1).

### 3.2. Effect of zileuton on the oxidative status of PSCs

**Fig. 2:**
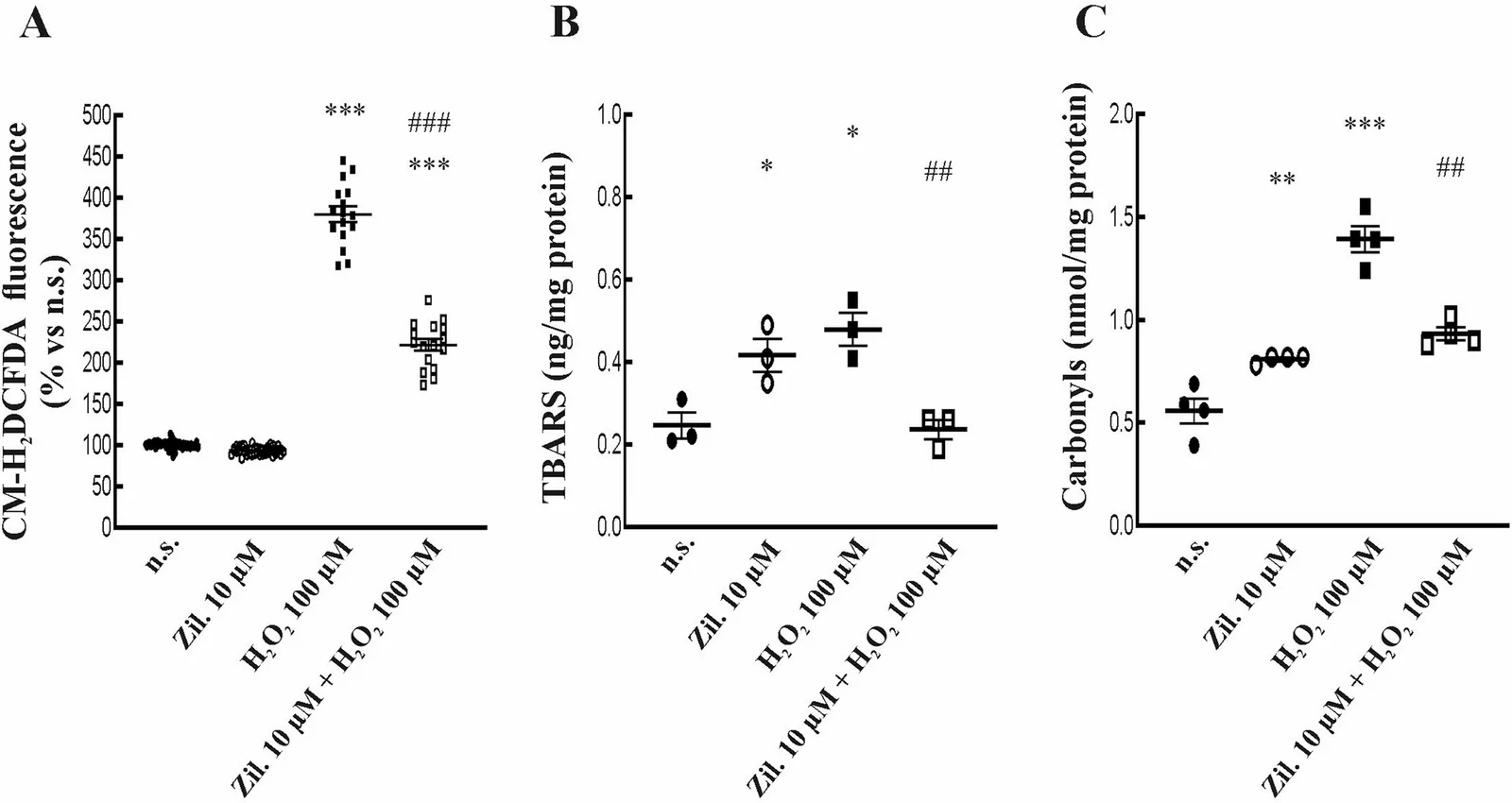
Effect of zileuton on the oxidative state of PSCs. (**A**) The effect of zileuton on the generation of ROS was tested. Cells were loaded with the red-ox-sensitive dye CM-H_2_DCFDA and were incubated during 1 h in the presence of 10 µM zileuton (Zil). As a control of oxidation, separated batches of cells were incubated during 1 h in the presence of 100 µM hydrogen peroxide (H_2_O_2_). A third group of cells was incubated during 5 min with zileuton (10 µM), prior to addition of H2O2 (100 µM). The cells were further incubated for 1 h. The graphs show the mean increase of dye-derived fluorescence expressed in percentage ± SEM with respect to non-treated cells (incubated in the absence of drugs). (**B**) The levels of TBARS and (**C**) protein carbonyls were studied as index of lipids and protein oxidation, respectively. The bars report the changes in the oxidation of lipids (TBARS) or proteins (carbonyls) in PSCs incubated for 4 h in the presence of zileuton (Zil, 10 M), H_2_O_2_ (100 µM) or a combination of them. The values were compared to those observed in non-treated cells (cells incubated in the absence of drugs). The bars show the mean increase in TBARS (ng/mg protein) or carbonyls (nmol/mg protein) ± S.E.M. with respect to non-treated cells (incubated in the absence of drugs). Results are representative of three independent experiments (n.s., non-treated cells; *, *P* < 0.05, **, *P* < 0.01 and ***, *P* < 0.001 vs. non-treated cells; ##, *P* < 0.01 and ###, *P* < 0.001 vs. H_2_O_2_)

LOX is involved in the generation of ROS, which occurs majorly via its metabolites, especially 5-LOX metabolites [21]. Moreover, we have shown in a former work that the oxidative state of PSCs is critical for their proliferation [5]. Thus, we evaluated the effect of zileuton on the generation of ROS. For this purpose, PSCs were loaded with the ROS-sensitive fluorescent dye CM-H_2_DCFDA. Thereafter, cells were incubated for 1 h in the presence of zileuton (10 µM). We chose this concentration of zileuton in view of the results shown above. In the presence of the 5-LOX inhibitor, no changes in dye-derived fluorescence were noted (94.43 ± 2.75, *n* = 5), compared to that observed in non-treated cells (100.00% ± 0.57, *n* = 5; Fig. 2A). As a control of oxidation, cells were challenged during 1 h in the presence of hydrogen peroxide (H_2_O_2_; 100 µM). Following the addition of the oxidant to the cells, a statistically significant increase in dye-derived fluorescence was observed (388.90% ± 15.70, *n* = 4; *P* < 0.001) compared to that observed in non-treated cells (100.00% ± 0.57, *n* = 5), reflecting an increase in oxidation (Fig. 2A). Next, we tested the combination of H_2_O_2_ (100 µM) plus zileuton (10 µM; 10 min. preincubation). After 1 h of incubation with the oxidant the detection of ROS was significantly diminished (218.20% ± 15.70, *n* = 4; *P* < 0.001) in comparison with the level of oxidation observed when H_2_O_2_ was applied alone (388.90% ± 15.70, *n* = 4; Fig. 2A).

In the next series of experiments, we studied the effect of zileuton on lipid peroxidation and protein oxidation. TBARS (Thiobarbituric Acid Reactive Substances) assay is a widely used laboratory method to measure malondialdehyde (MDA), a key biomarker of lipid peroxidation and oxidative stress. Treatment of cells for 4 h with zileuton (10 µM) evoked a statistically significant increase in the level of TBARS (0.42 ng/mg protein ± 0.04, *n* = 3) in comparison with non-treated cells (incubated in the absence of zileuton) (0.25 ng/mg protein ± 0.03, *n* = 3, *P* < 0.05; Fig. 2B). When cells were incubated in the presence of H_2_O_2_ (100 µM) a statistically significant increase in the detection of TBARS was noted (0.48 ng/mg protein ± 0.05, *n* = 3) in comparison with non-treated cells (incubated in the absence of zileuton; *P* < 0.05). In turn, preincubation of PSCs with zileuton (4 h, 10 µM) prior treatment with H_2_O_2_ (100 µM; 1 h treatment) significantly diminished oxidation of lipids (0.24 ng/mg protein ± 0.02, *n* = 3) with respect to that evoked by H_2_O_2_ (*P* < 0.01; Fig. 2B).

Similar effects were noted when the levels of protein oxidation were studied. Treatment of cells for 4 h with zileuton (10 µM) evoked a statistically significant increase in the level of carbonyls (0.81 nmol/mg protein ± 0.01, *n* = 3) in comparison with non-treated cells (incubated in the absence of zileuton) (0.55 nmol/mg protein ± 0.06, *n* = 3, *P* < 0.01; Fig. 2C). When cells were incubated in the presence of H_2_O_2_ (100 µM) a statistically significant increase in the detection of carbonyls was noted 1.39 nmol/mg protein ± 0.08, *n* = 3) in comparison with non-treated cells (incubated in the absence of zileuton) (*P* < 0.001; Fig. 2C). Finally, preincubation of PSCs with zileuton (4 h, 10 µM) prior treatment with H_2_O_2_ (100 µM; 1 h treatment) significantly diminished oxidation of proteins (0.93 nmol/mg protein ± 0.04, *n* = 3) with respect to that evoked by H_2_O_2_ (*P* < 0.01; Fig. 2C).

### Effect of zileuton on PSC's antioxidant defenses

**Fig. 3:**
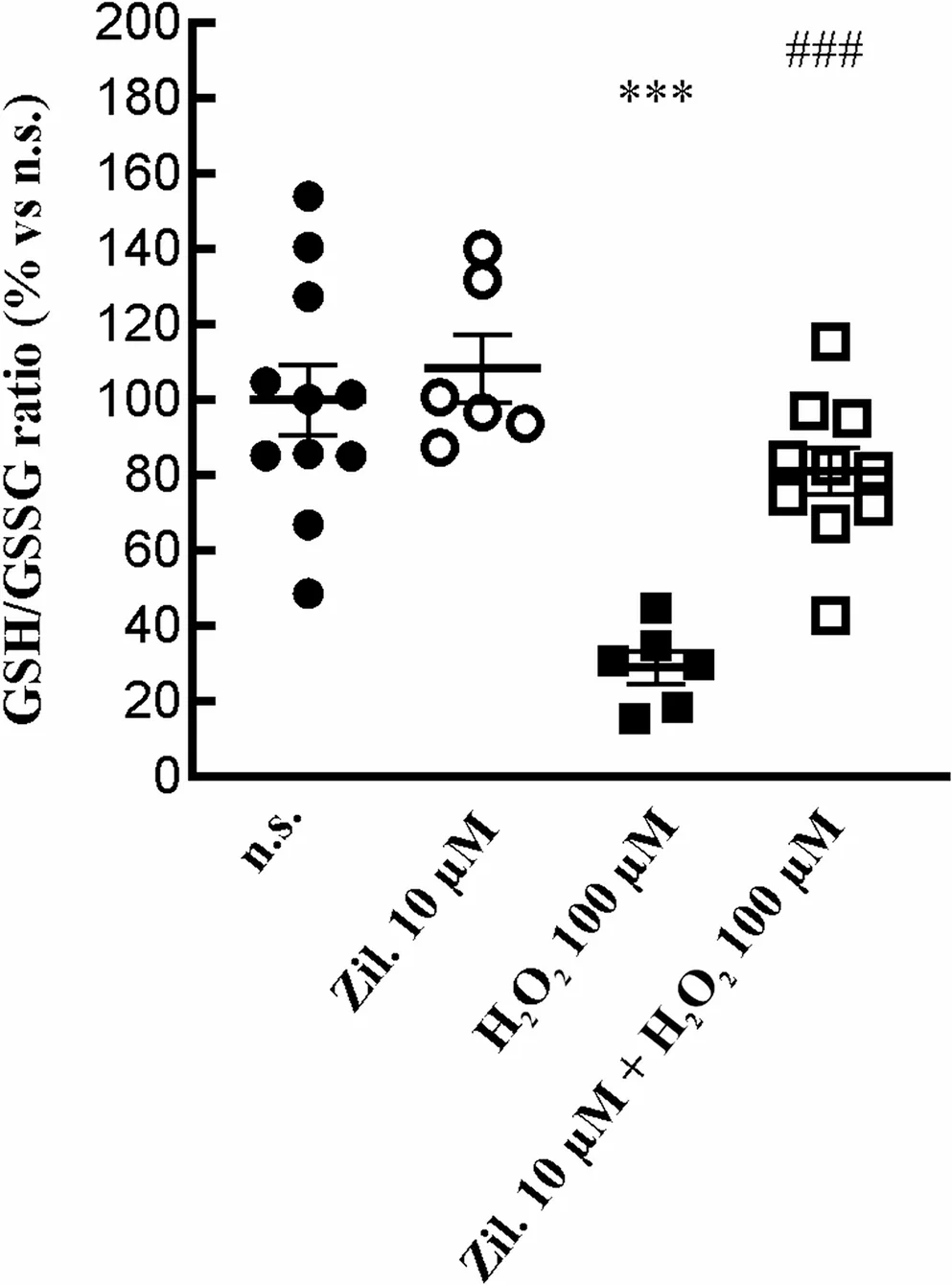
Effect of zileuton on glutathione levels. The effect of zileuton on the levels of glutathione was studied. PSCs were incubated during 4 h in the absence (non-treated), in the presence of zileuton (Zil., 10 µM) or with zileuton (10 µM) plus H_2_O_2_ (100 µM). The levels of GSH and GSSG were determined. The graph shows the mean increase in GSH/GSSG ratio expressed in percentage ± S.E.M. with respect to non-treated cells (incubated in the absence of drugs) (n.s., non-treated cells; ***, *P* < 0.001 vs. non-treated cells; ###, *P* < 0.001 vs. H_2_O_2_). The experiments shown are representative of three different preparations

Glutathione (gamma-glutamyl-cysteinyl-glycine; GSH) is a low-molecular-weight thiol and represents an important antioxidant defense against oxidative stress [22]. Because we had observed a modulation of ROS production in the presence of the zileuton, we next tested its effect on the glutathione system in PSCs. Thus, PSCs were incubated during 4 h in the presence of the 5-LOX inhibitor (10 µM) and the levels of GSH and GSSG were analyzed. Zileuton alone did not induce significant changes in GSH/GSSG ratio (108.40% ± 8.93, *n* = 3) compared to the level noted in non-stimulated cells (100.00% ± 9.04, *n* = 3; Fig. 3). When cells were challenged in the presence of H_2_O_2_ (100 µM) for 4 h, a statistically significant decrease in the GSH/GSSG ratio was noted (28.84% ± 4.39, *n* = 3, *P* < 0.001), compared to that observed in non-treated cells (100.00% ± 9.04, *n* = 3; Fig. 3). Additionally, we tested the combination of H_2_O_2_ (100 µM; 10 min. treatment) plus zileuton (10 µM; 4 h preincubation preincubation). Following treatment of the cells, the GSH/GSSG ratio augmented (84.16% ± 24.94, *n* = 3, *P* < 0.05) in comparison with the level observed when H_2_O_2_ was applied alone (28.84% ± 4.39, *n* = 3; Fig. 3).

**Fig. 4:**
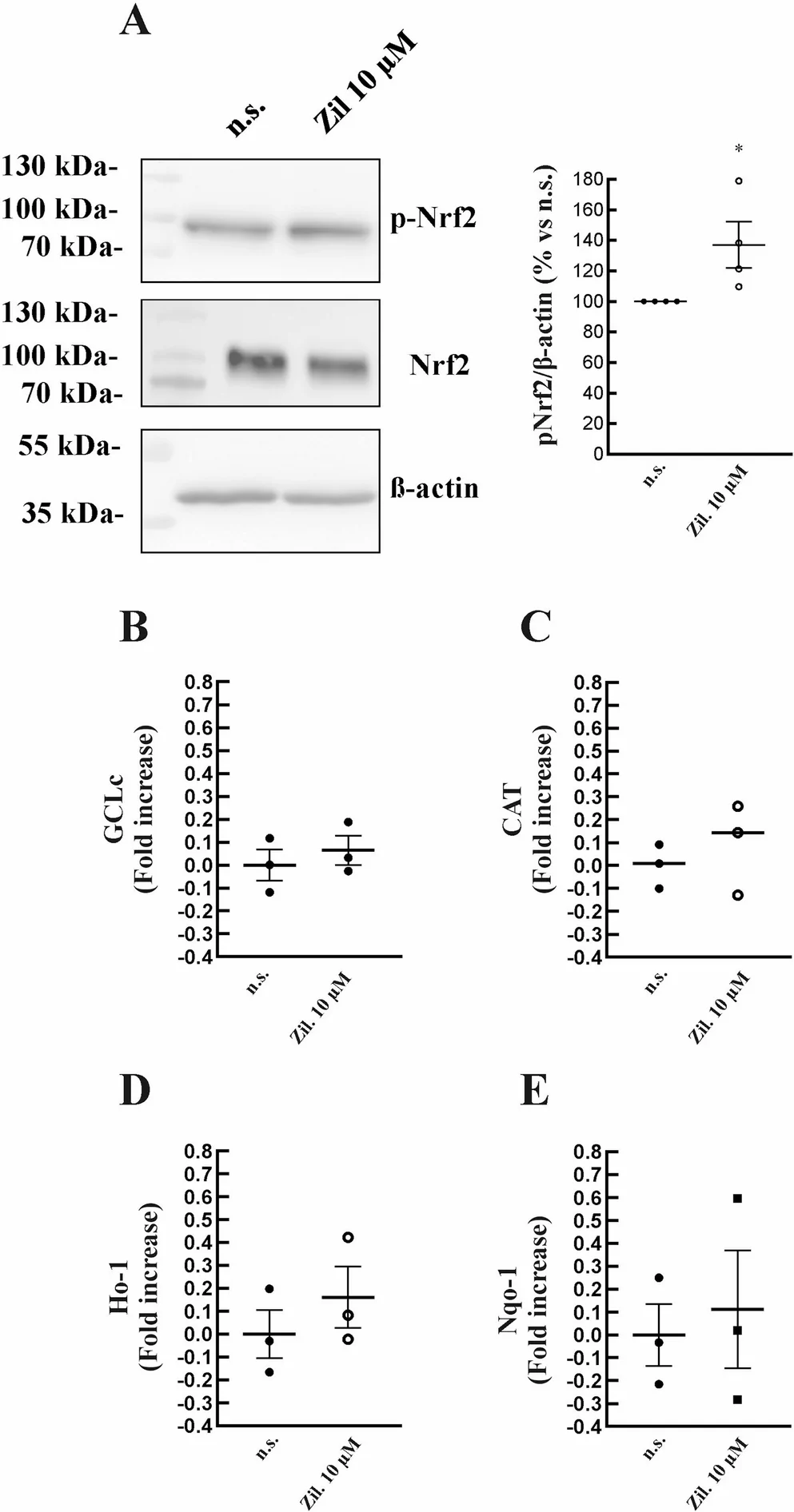
Effect of zileuton on Nrf2 phosphorylation and on Nrf2-related antioxidant enzymes. PSCs were incubated during 4 h in absence (non-treated) or in the presence of zileuton (Zil; 10 µM). The figure shows a representative blot displaying the effect of zileuton on the phosphorylation of Nrf2 (**A**), which was evaluated with a specific antibody p-Nrf2-Ser40). The levels of β-actin were employed as controls to ensure equal loading of proteins. The bars show the quantification of protein expression. Values are shown as the mean ± S.E.M. of normalized values expressed as % of phosphorylation (Nrf2) with respect to non-treated cells (n.s., non-treated cells; *, *P* < 0.05; vs. non-treated cells). The experiments shown are representative of four to five different preparations. (B-E) RT-qPCR analysis was used to study the effect of zileuton on the levels of various Nrf2-dependent antioxidant enzymes: cysteine ligase-catalytic subunit (**B**; GClc), catalase (**C**; CAT), heme-oxygenase-1 (**D**; Ho-1) and NAD(P)H quinone oxidoreductase 1 (E; Nqo1). *Gapdh* mRNA was used for normalization. The graphs show the fold increase of mRNA levels ± S.E.M. of each protein relative to non-stimulated cells (incubated under hypoxia but in the absence of melatonin). Three different preparations were used (n.s., non-stimulated cells; Zil., zileuton)

Nrf2 is a transcription factor that is majorly involved in the regulation of cellular redox homeostasis [23]. We have previously reported its implication in the modulation of PSCs redox state [5, 24]. Because the results shown above indicate that zileuton induced changes in the redox status of PSCs, we thus decided to study whether the activation of Nrf2 also occurred. For this purpose, PSCs were incubated for 4 h in the absence (non-treated cells) or in the presence of zileuton (10 µM) and the phosphorylation of this transcription factor was detected by Western blotting. Following incubation of PSCs in the presence of the 5-LOX inhibitor a slight, but statistically significant, increase in the phosphorylation of Nrf2 was observed (137.00% 15.13, *n* = 4), compared to the level noted in non-treated cells (100.01% ± 24.94, *n* = 5, *P* < 0.05; Fig. 4A).

To study whether zileuton could stimulate the transcriptional activation of antioxidant enzymes through the activation of Nrf2, RT-qPCR of the relative mRNA abundance was performed. Results reveal that the 5-LOX inhibitor (4 h incubation; 10 µM) evoked a slight, although not statistically significant, increase in the expression of GCLc, CAT, Ho-1 and Nqo-1 compared to the levels noted in non-treated cells (Fig. 4B-E).

**Fig. 5:**
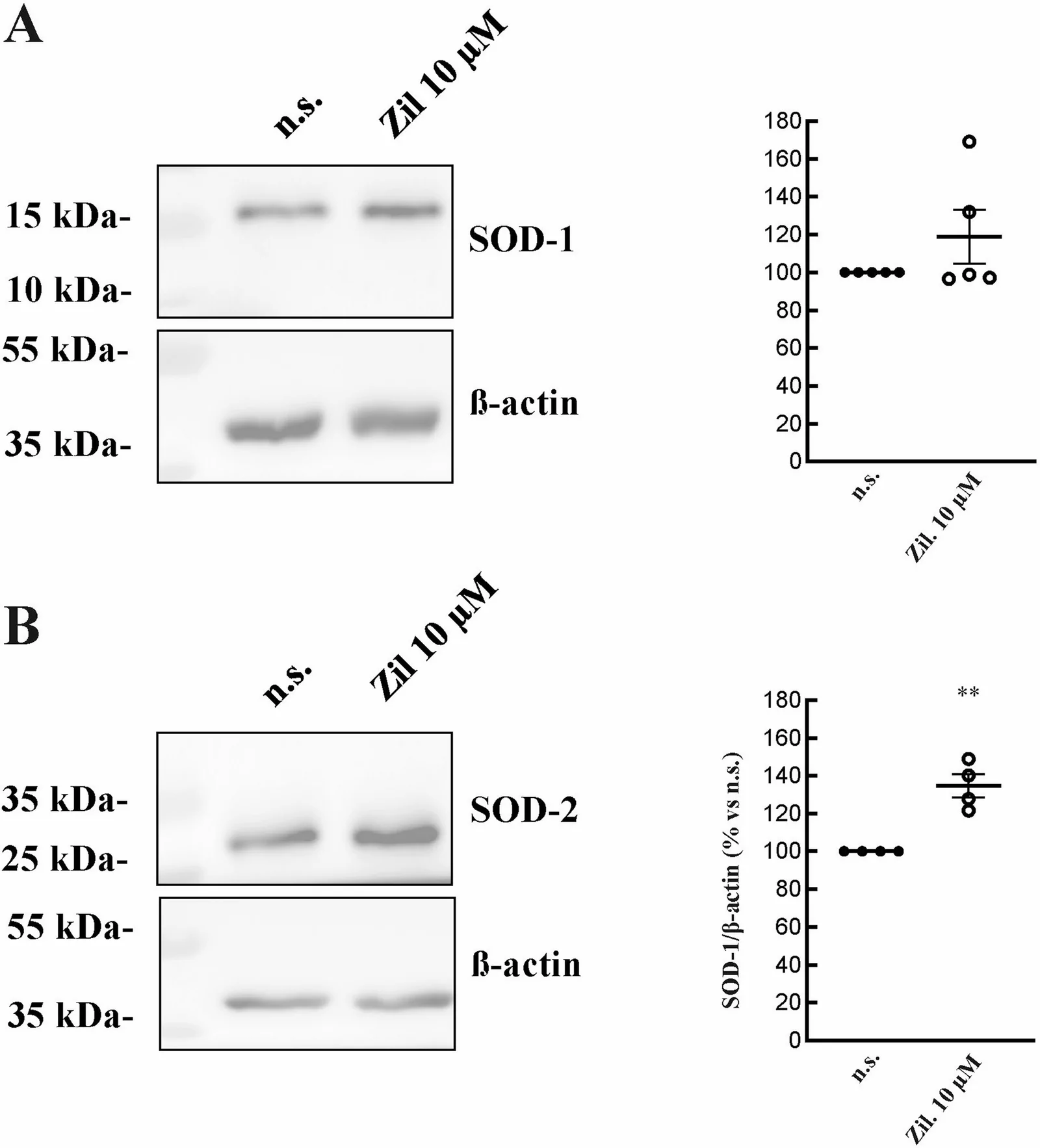
Effect of zileuton on the expression of SOD-1 and SOD-2. PSCs were incubated during 4 h in absence (non-treated) or in the presence of zileuton (Zil; 10 µM). The figure shows representative blots displaying the effect of zileuton on the expression of the antioxidant enzymes SOD-1 (**A**) and SOD-2 (**B**), which were evaluated with specific antibodies. The levels of β-actin were employed as controls to ensure equal loading of proteins. The graphs show the quantification of protein expression. Values are shown as the mean ± S.E.M. of normalized values expressed as % of protein expression (SOD-1 and SOD-2) with respect to non-treated cells (n.s., non-treated cells; **, *P* < 0.01 vs. non-treated cells). The experiments shown are representative of four to five different preparations

Superoxide dismutases (SODs) are a clash of enzymes that are involved in the cellular defense against ROS [25] and are included within the antioxidant enzymes depending on Nrf2-regulated redox genes [26]. In PSCs, SOD-1 and SOD-2 are involved in the antioxidant response [4, 5]. At this point, we were interested in analyzing whether inhibition of 5-LOX by zileuton exerted any effect on SODs expression. For this purpose, PSCs were incubated during 4 h in absence (non-treated cells) or in the presence of zileuton (10 µM) and the protein levels of the enzymes were studied by Western blotting. The results show that zileuton increased the expression of both SOD-1 (118.70% ± 14.25, *n* = 5) and SOD-2 134.70% ± 6.12, *n* = 5, *P* < 0.05), compared with the level noted in non-treated cells (100.01% ± 0.01, *n* = 5) (Fig. 5A and B).

### Effect of zileuton on ferroptosis

Ferroptosis is a regulated cell death mechanism that was originally detected in cancer cells. It is also involved in the development of pancreatitis [27]. At this point, we decided to incubate PSCs in the absence or in the presence of zileuton, alone or in combination with drugs with known effects on ferroptosis. Then, cell viability was studied.

**Fig. 6:**
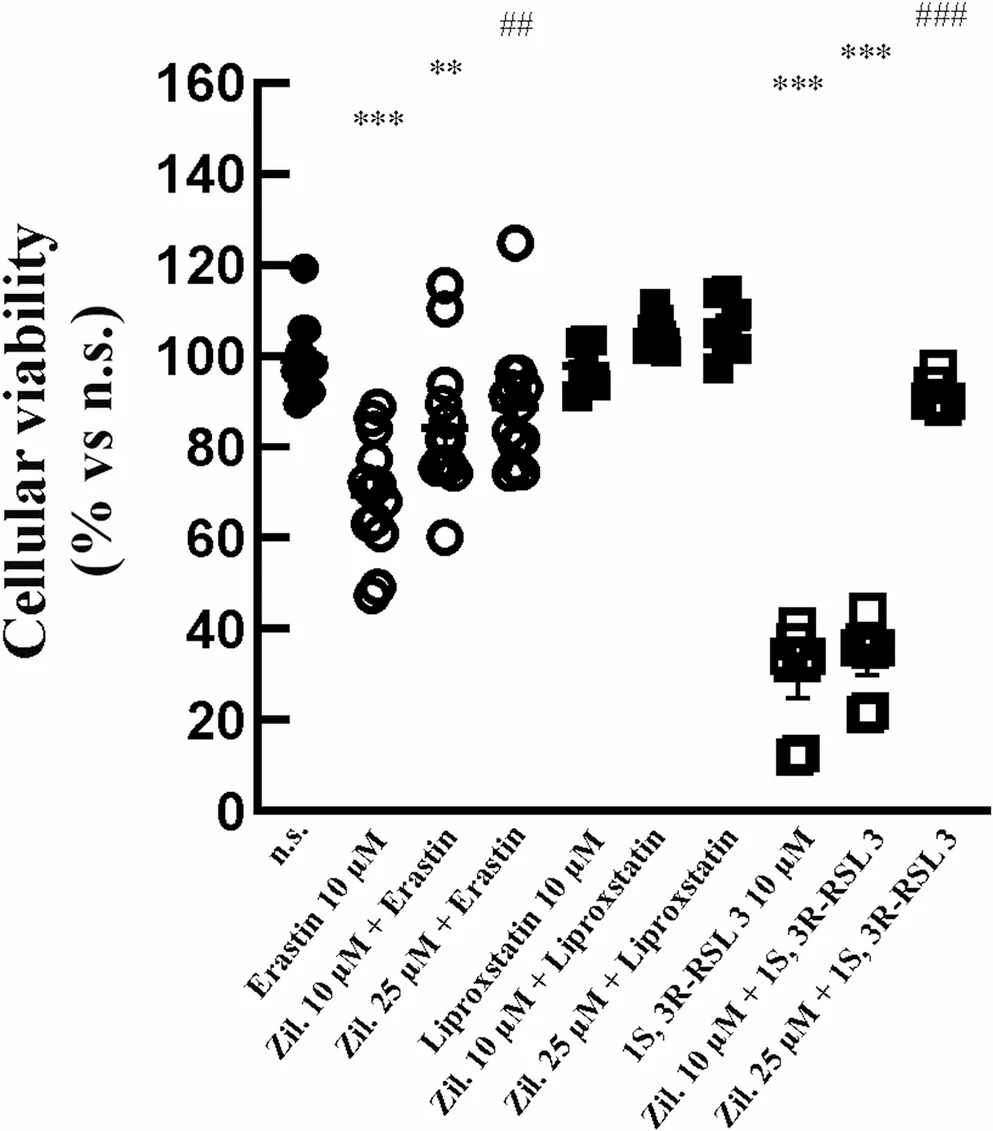
Effect of zileuton on ferroptosis. Different batches of cells were incubated during 24 h in absence (non-treated), in the presence of ferroptosis-related drugs erastin, liproxstatin or 1 S,3R-RLS alone or in combination with zileuton (Zil., 10 µM or 25 µM). Viability of cells subjected to treatments with drugs was compared with that of cells incubated its absence (non-treated cells), which was considered 100%. Additionally, viability of cells treated with the combination of zileuton (10 µM or 25 µM) plus the inductor or inhibitor of ferroptosis was compared to that of cells treated with the ferroptosis inductor or inhibitor alone. The graph shows the mean increase in cellular viability expressed in percentage ± S.E.M. with respect to cells incubated in the absence of drugs (n.s., non-treated cells; **, *P* < 0.01; ***, *P* < 0.001 vs. non-treated cells). Comparisons of the effects of the combination of treatments also were performed (##, *P* < 0.01 and ###, *P* < 0.001 vs. respective ferroptosis inductor/inhibitor alone). The experiments shown are representative of three to four different preparations

For this purpose, PSCs were challenged in the presence of erastin, a well know inductor of ferroptosis [28]. Incubation of PSCs for 24 h in the presence of erastin (10 µM) a statistically significant drop in cell viability was observed (70.11% ± 2.89, *n* = 4), compared to viability of non-treated cells (*P* < 0.001). Thereafter, cells were incubated in the presence of a combination of erastin plus zileuton (10 µM or 25 µM), the desired concentration of zileuton being applied 4 h prior to addition of erastin. After treatment of cells with zileuton plus erastin for 24 h, an increase in cellular viability was noted at 10 µM zileuton (82.88% ± 4.43, *n* = 4, statistically non-significant) and 25 µM zileuton (87.16% ± 3.23, *n* = 4, *P* < 0.01), compared to that observed in cells treated with erastin alone (70.11% ± 2.89, *n* = 4) (Fig. 6).

The next series of experiments were preformed employing liproxstatin-1, a selective inhibitor of ferroptosis [29]. First, the cells were incubated for 24 h with the ferroptosis inhibitor (10 µM). In the presence of liproxstatin-1, no statistically significant changes were noted compared to viability of non-treated cells (94.98% ± 2.74, *n* = 3 vs. 100.00% ± 2.01, *n* = 4, for liproxstatin-1 and non-treated cells, respectively). Afterwards, cells were incubated in the presence of a combination of liproxstatin plus zileuton (10 µM or 25 µM), the desired concentration of zileuton being applied 4 h prior to addition of liproxstatin. No changes in cellular viability were observed when PSCs were incubated in the presence of 10 µM plus liproxstatin (104.50% ± 1.54, *n* = 3) or 25 µM zileuton plus liproxstatin (104.7% ± 2.27, *n* = 3), compared to viability of cells incubated with liproxstatin-1 alone (94.98% ± 2.74, *n* = 3) (Fig. 6).

In a last step, PSCs were treated with 1 S,3R-RLSL 3, an inhibitor of glutathione peroxidase 4 (GPX4). This enzyme exerts antioxidant effects via neutralization of lipid peroxides in the presence of GSH. 1 S,3R-RLSL 3 is thus considered and inductor of ferroptosis [30]. In this set of experiments, PSCs were incubated first for 24 h with 1 S,3R-RLSL 3 (10 µM). In the presence of the ferroptosis inductor a statistically significant decrease in cellular viability was detected (23.33% ± 4.25, *n* = 3) compared to that noted in non-treated cells (100.00% ± 2.01, *n* = 4; *P* < 0.001). Thereafter, cells were incubated in the presence of a combination of 1 S,3R-RLSL 3 plus zileuton (10 µM or 25 µM), the desired concentration of zileuton being applied 4 h prior to addition of the GPX4 inhibitor. No statistically significant difference in cellular viability was observed in the viability of cells incubated in the presence of 10 µM zileuton plus 1 S,3R-RLSL 3 (29.24% ± 3.02, *n* = 3), compared to viability of cells incubated with 1 S,3R-RLSL 3 alone (23.33% ± 4.25, *n* = 3). However, cell viability was restored significantly when PSCs were treated with the combination of 25 µM zileuton plus the GPX4 inhibitor (87.21% ± 2.38, *n* = 3) in comparison to viability of cells treated with 1 S,3R-RLSL 3 alone (*P* < 0.001) (Fig. 6).

### Effect of zileuton on mitochondrial activity

The compound AlamarBlue^®^ is a ready-to-use resazurin-based reagent that functions as cell health indicator by using the reducing power of living cells. Upon entering living cells, resazurin is reduced to resorufin, a compound that is red in color and highly fluorescent. Resazurin is used as an oxidation-reduction (REDOX) indicator that undergoes colorimetric change in response to cellular metabolic reduction. Traditionally, it is used to quantitatively measure cellular viability. Nevertheless, in the short term (few hours) it can also be used to monitor mitochondrial activity, because it detects the level of oxidation during respiration [31].

**Fig. 7:**
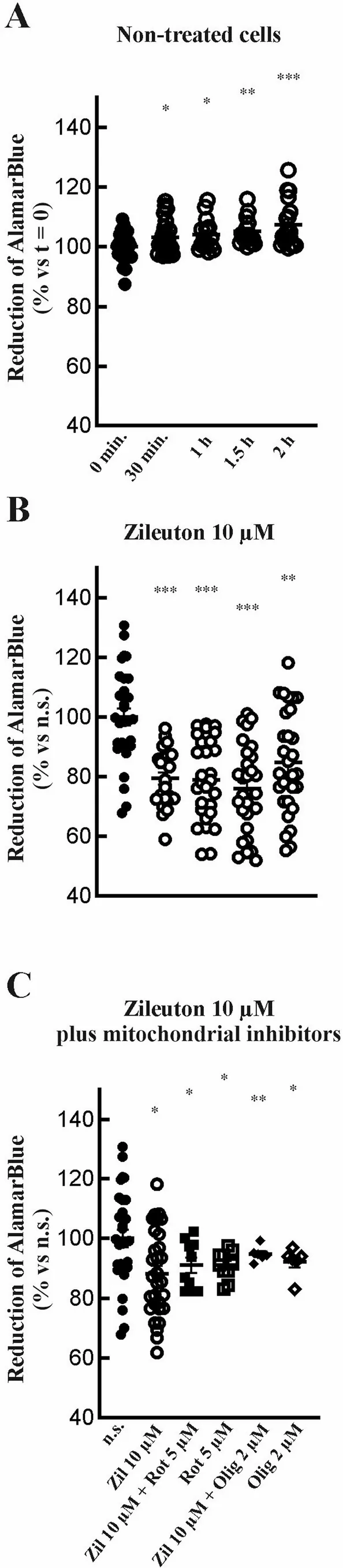
Effect of zileuton on mitochondrial reduction of AlamarBlue. (**A**) PSCs were incubated in the presence of the resazurin-based compound AlamarBlue^®^ (10% v/v) and in the absence of zileuton (Zil.) in the incubation medium (non-treated cells). Fluorescence data were collected at the beginning of the incubation period (time = 0 min.) and, thereafter, every 30 min., for 2 h. Resazurin is reduced to resorufin by the cells. Resofurin is red in color and highly fluorescent, and can be used as a REDOX indicator, reflecting cellular metabolic reduction. The graph shows the mean reduction of resazurin expressed in % ± S.E.M. with respect to the values observed at the beginning of the incubation period (*, *P* < 0.05; **, *P* < 0.01; and ***, *P* < 0.001 vs. absorbance detected at the beginning of the incubation period). (**B**) PSCs were incubated with zileuton (Zil., 10 µM) in the presence of the resazurin-based compound AlamarBlue^®^ (10% v/v) in the incubation medium, for 2 h. Fluorescence data were collected after the addition of zileuton every 30 min for 2 h (**, *P* < 0.01; and ***, *P* < 0.001 vs. absorbance detected in non-treated cells, n.s.). (**C**) PSCs were incubated in the presence of the resazurin-based compound AlamarBlue^®^ (10% v/v) and treated with zileuton (Zil., 10 µM), the mitochondrial inhibitor rotenone (Rot., 5 µM) or oligomycin (Olig., 2 µM) alone or in combination (Zil.+Rot. or Zil-+Olig.), for 2 h. Fluorescence data were collected after the addition of zileuton every 30 min for 2 h. The experiments shown are representative of three different preparations

First, PSCs were incubated in the presence of AlamarBlue^®^ (10% v/v) in the incubation medium, for different time periods ranging from 0 to 2 h. Data were collected every 30 min. Reduction of resazurin was increased in non-treated cells (which were incubated in the absence of zileuton) at 30 min. (103.30% ± 1.09, *n* = 3, *P* < 0,05), 1 h (104.00% ± 1.37, *n* = 3, *P* < 0.05), 1,5 h (105.20% ± 1.25, *n* = 3, *P* < 0.01) and 2 h (107.40% ± 1.76, *n* = 3, *P* < 0.001), with respect to the value noted at the beginning of the incubation period (time = 0 min.) (100.00% ± 0.86, *n* = 3) (Fig. 7A).

Separate batches of PSCs were incubated with zileuton (10 µM) in the presence of AlamarBlue^®^ (10% v/v) in the incubation medium, for 2 h. Data were collected every 30 min. In the presence of the 5-LOX inhibitor, a drop in the reduction of resazurin was noted at 30 min. (79.46% ± 2.01, *n* = 3, *P* < 0.001), 1 h (78.99% ± 2.56, *n* = 3, *P* < 0.001), 1.5 h (76.06% ± 2.74, *n* = 3, *P* < 0.001) and 2 h of incubation (84.88% ± 3.23, *n* = 3, *P* < 0.001) with respect the value observed in non-treated cells, which were incubated in the absence of the 5-LOX inhibitor (100.00% ± 2.89, *n* = 3) (Fig. 7B).

To check mitochondrial reduction of AlamarBlue^®^, PSCs were preincubated for 5 min. in the presence of the mitochondrial inhibitors rotenone (5 µM), an inhibitor of mitochondrial complex I, or oligomycin (2 µM), an inhibitor of the ATP synthase, and thereafter were further incubated with zileuton (10 µM). In the presence of the mitochondrial inhibitors plus zileuton, no statistially significant differences were observed with respect to the values obtained in the presence of the 5-LOX inhibitor alone (91.03 ± 2.548, *n* = 3 for rotenone; 91.06 ± 1.514, *n* = 3, for oligomycin; and 88.23 ± 2.660, *n* = 3 for zileuton; Fig. 7C).

**Fig. 8:**
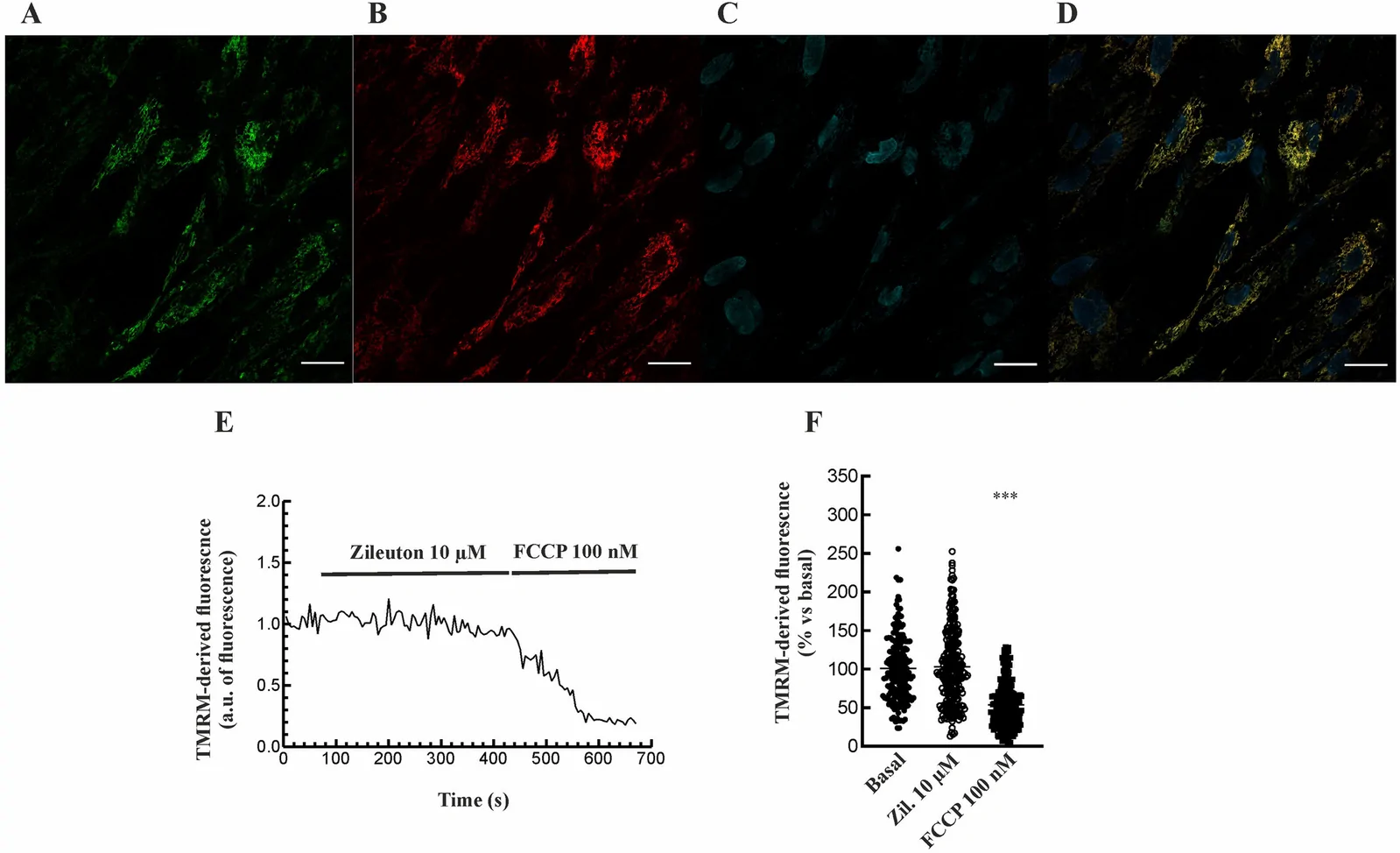
Effect of zileuton on mitochondrial membrane potential. Confocal fluorescence images of PSCs loaded with Mitotracker™ Green FM (**A**), TMRM (**B**) and Hoechst (**C**). Merged images are shown in **D**. The images show the distribution of mitochondria within the cells (scale bar, 20 μm). Pictures are representative of three different preparations. (**E**) Confocal microscopy was used to monitor time-course of changes in TMRM-derived fluorescence. Small round fluorescent areas, consistent with mitochondria, were selected to monitor fluorescence. PSCs, loaded with the fluorescent probe TMRM (50 nM), were incubated with zileuton (10 µM) for 5 min. Afterwards, the mitochondrial uncoupler FCCP (100 nM) was added to the extracellular medium, and cells were further incubated for 5 min. The graph shows a representative time-course of changes in TMRM-derived fluorescence of one individual mitochondrial area. Data are expressed as absolute values of fluorescence normalized to basal (prestimulation) level. (**F**) An epifluorescence inverted microscope was used to detect changes in TMRM-derived fluorescence of areas considered individual mitochondria. The graph shows the mean values of TMRM-derived fluorescence achieved after treatments with zileuton (Zil., 10 µM) and FCCP (100 nM). Results are expressed as the mean ± SEM (n) of fluorescence values expressed as % vs. fluorescence noted at the beginning of the experiment (***, *P* < 0.001 vs. fluorescence detected at the beginning of the incubation). The experiments shown are representative of four different preparations

A different set of experiments was dedicated to monitorization of changes in Ψm. For this purpose, cells were loaded with TMRM, a specific dye that is sensitive to changes in Ψm. To confirm accumulation of TMRM within mitochondria, cells were coloaded with MitoTracker™ Green FM, a specific marker of mitochondria [32]. Nuclei were marked using Hoechst 33,342. Confocal microscopy revealed a mitochondrial network with a bright, intense green (Fig. 8A, MitoTracker™ Green FM) and red (Fig. 8B, TMRM) fluorescence. Nuclei appear in blue (Fig. 8C; Hoechst 33342). A merged image of all three dyes is shown in Fig. 8D.

In separate sets of experiments, PSCs, loaded with TMRM, were challenged with 10 µM zileuton for 5 min. In the presence of the 5-LOX inhibitor we could not detect significant changes in Ψm with respect to the value observed at the beginning of the experiment. However, after the addition of the mitochondrial uncoupler FCCP (100 nM) a drop in Ψm was detected with respect to the value noted at the beginning of the experiment (Fig. 8E). Quantification of fluorescence revealed no statistically significant differences in Ψm in the presence of zileuton compared to the basal (prestimulation) values (*n* = 4 experiments/255 mitochondrial areas/60 cells). However, statistically significant differences were detected in Ψm after addition of FCCP with respect to the values observed at the beginning of the experiment (*n* = 4 experiments/238 mitochondrial areas/60 cells) (Fig. 8F).

**Fig. 9:**
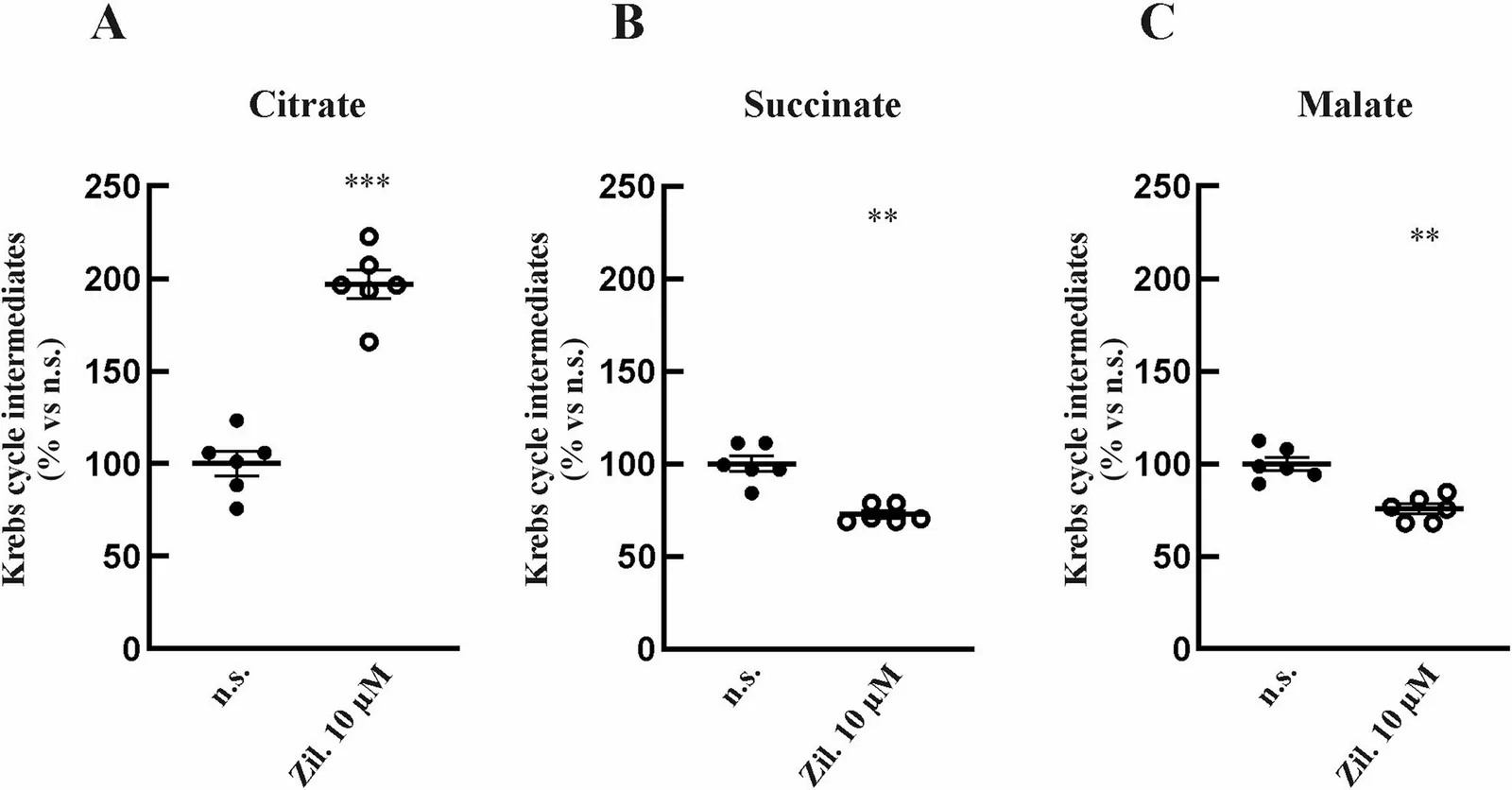
Effect of zileuton on Krebs cycle intermediates. PSCs were incubated in the presence or in the absence of zileuton (Zil.; 10 µM) in the incubation medium for 4 h. Then cells were harvested and lysed, and the levels of various intermediates of the Krebs cycle were analyzed. The graph shows the levels detected of citrate (**A**), succinate (**B**) and malate (**C**) in cells incubated in the presence or in the absence (n.s., non-treated cells) of the 5-LOX inhibitor. Results are expressed as the mean ± SEM (n) of metabolite values expressed as % vs. the level noted in non-treated cells (**, *P* < 0.01 and ***, *P* < 0.001 vs. non-treated cells). The experiments shown are representative of three different preparations

Further characterization of zileuton on mitochondrial activity was performed by studying its effect on the Krebs cycle, which fuels the electron transport chain by stripping high-energy electrons from carbon molecules. PSCs were incubated for 4 h in the presence of zileuton (10 µM). In the presence of the 5-LOX inhibitor a statistically significant increase in the detection of citrate was observed with respect to non-treated cells, incubated in the absence of zileuton (197,04% ± 1,38, *n* = 6 vs. 100,00% ± 1,48, *P* < 0,001). On the contrary, the levels of succinate (72,68% ± 2,73, *n* = 6, *P* < 0,01) and malate (75,64% ± 2,31, *n* = 6, *P* < 0,01) were decreased in comparison to the levels noted in non-treated cells (100,00 ± 1,48) (Fig. 9).

## Discussion

LOX comprises a family of enzymes responsible for AA metabolization to derivatives, generally termed eicosanoids. Cyclooxygenase and cytochrome P450 are additional pathways that participate in the conversion of AA to its corresponding derivatives [33]. AA is a cellular membrane lipid constituent that can be released, majorly, by PLA2 family of enzymes [34]. Eicosanoids are highly bioactive lipid compounds that act on many cell types and modulate a wide array of cellular and molecular processes, either in health or disease. Among them, inflammation is a physiological process that, under normal conditions, eradicates the source of damage and restores tissue function. However, excessive, uncontrolled or prolonged inflammation is deleterious for cellular physiology and represents the basis of chronic pathologies such as pancreatitis, diabetes or cancer, among others [35]. Therefore, inhibition of AA metabolization has been signaled as a probable point of action for the treatment of inflammation and, additionally, for treatment of cancer [36, 37]. In this line, the inhibition of LOX has been suggested as a valuable tool for the treatment of disease. Concretely, experimental data have suggested a correlation between tumoral cells viability and LOX gene expression, especially, 5-LOX [37, 38].

When activated, PSCs proliferate and contribute to the development of fibrotic tissue, characteristic of inflamed pancreas or tumoral tissue developing in the gland [39]. Maneuvers to handle the stroma have been suggested as tools to control the growth of tumoral tissue and ease treatments [40, 41].

A major contributor to the proliferation of PSCs is their oxidative state. As such, a certain level of oxidative stress drives PSCs proliferation and their contribution to stroma [5]. Conversely, excessive or uncontrolled oxidative stress is deleterious for pancreatic cellular physiology and can induce cell death [42]. Therefore, control of PSCs oxidative state is pivotal to their physiology. Previous work, carried out in our laboratory, has shown interesting results which suggest that modulation of PSCs antioxidant defenses is valuable to control their proliferation [4, 43, 44].

As mentioned above, the inhibition of LOX has been signaled as a valuable tool for the treatment of disease. However, despite its prominent role in pancreatitis and in the development and growth of pancreatic tumors, studies on the effect of LOX inhibition on the physiology of PSCs are currently lacking.

In the present study, we were interested in studying the actions of zileuton, a 5-LOX inhibitor [9], on PSCs physiology. The concentrations of zileuton that we have used in our study fall within the range of those successfully used by others in former studies [45–47].

Our results have shown that the inhibition of 5-LOX by zileuton diminished oxidation induced by H_2_O_2_. This was observed as a decrease in CM-H_2_DCFDA-derived fluorescence and a drop in lipid and proteins oxidation. Applied alone, zileuton did not induce detectable changes in ROS production, although this molecule induced an increase in lipid peroxidation. Interestingly, upon treatment of cells with the 5-LOX inhibitor, increases in the ratio of GSH/GSSG, in the phosphorylation of Nrf2 and in the expression related antioxidant enzymes were observed. All these observations suggest that zileuton-induced lipid and protein oxidation may act as a trigger to initiate a cell-protective antioxidant response that includes the activation of Nrf2 and its dependent antioxidant systems, such as GCLc, CAT, Ho-1, Nqo-1, SOD-1/2 or the glutathione system. The antioxidant effect of zileuton is more prominent in the context of strong oxidation, as is the case of H_2_O_2_ treatment. Under the effect of the latter, zileuton reduces both ROS production and protein and lipid oxidation.

Ferroptosis is death/survival mechanism that involves oxidative injury to cellular components based on iron-dependent lipid peroxidation [48]. Because we had observed effects on the general oxidative status in the presence of zileuton, it was worth studying its effect of ferroptosis. Our results have shown that zileuton attenuated the effect of ferroptosis inducers on cell viability, consistent with ferroptosis inhibition. Moreover, zileuton alone did not alter PSCs viability significantly, i.e., no deleterious actions of the 5-LOX inhibitor on cell viability were observed. Therefore, zileuton appears as a protector against ferroptosis. The failure of 10 µM zileuton to rescue cell viability compared to the rescue by 25 µM zileuton under GPX4 inhibition (1 S,3R-RLSL 3) may stem from the severity of the oxidative insult and the concept of an enzymatic and metabolic threshold. Because RSL3 inflicts much more severe and direct ferroptotic stress than erastin, a higher threshold of protection is required to rescue the cells. At 10 µM, 5-LOX may be only partially inhibited. When GPX4 is functional or only partially challenged (as with erastin), 10 µM zileuton provides a minor protective trend. However, when GPX4 is completely blocked by RSL3, the remaining uninhibited 5-LOX activity could continue to generate lipid hydroperoxides. Because GPX4 cannot clear them, lipid ROS would accumulate above a certain threshold, leading to cell death. At 25 µM, zileuton achieves sufficient concentration to suppress 5-LOX activity below the critical generation flux. Beyond simple enzyme inhibition, the jump in cell viability observed between 10 µM and 25 µM zileuton, suggests a threshold effect involving secondary antioxidant mechanisms, that cross the threshold required to activate Nrf2. These observations agree with the antioxidant response noted in the presence of the 5-LOX inhibitor. Furthermore, ferroptosis is closely related to mitochondrial activity [48] and oxidative metabolism can cause lipid peroxidation in the execution of ferroptosis [49]. Thus, it was of interest to study whether zileuton exerted any effect on mitochondrial metabolic activity that might be related to its protective action against ferroptosis.

Interestingly, in the presence of zileuton, a drop (but not a complete inhibition) in the mitochondrial reduction of resazurin was noted, ranging a 20% approximately. Conversely, the mitochondrial reduction of resazurin was slightly increased in non-treated cells, i.e., incubated in the absence of zileuton (ranging a 7% approximately). The results obtained in the presence of mitochondrial inhibitors signal that AlamarBlue^®^ is majorly reduced within the mitochondria, therefore supporting our observations for a mitochondrial regulation by zileuton. Moreover, in the presence of zileuton, we could not detect changes in Ψm that could account for an impairment of mitochondrial physiology. Additionally, changes in the levels of intermediate metabolites of the Krebs cycle were detected. Zileuton acts as an effective ferroptosis inhibitor and triggers a protective mitochondrial metabolic adjustment, all without inducing cytotoxicity or altering cellular bioenergetic integrity. The 5-LOX inhibitor may be inducing a state of metabolic modulation or quiescence in which the cell reduces its basal metabolic activity while maintaining the functional integrity of its mitochondria, which in turn translates into robust cytoprotection against ferroptosis. The observation that the mitochondrial membrane potential remains unchanged confirms that zileuton at this concentration neither depolarizes nor uncouples mitochondria. The core respiratory machinery may maintain its proton gradient intact, and the mitochondria remain structurally sound and viable. The changes observed in the relative levels of metabolic intermediates indicate a deceleration in the flux rate of the Krebs cycle: the concurrent decrease in succinate and malate suggests lower carbon input into the intermediate/late stages of the cycle or a slowdown of key dehydrogenases. Thus, the cell produces and consumes these intermediates at a slower rate, reducing overall metabolic turnover without depleting the initial citrate pool. Consequently, the reduced AlamarBlue signal that we have observed is not a cytotoxicity artifact, but rather a direct reflection of the lower turnover rate in the Krebs cycle; as metabolic flux toward succinate and malate decreases, the rate of reducing equivalent generation declines, secondarily reducing the conversion of resazurin to resorufin. Zileuton, therefore, may place cells in a state of partial metabolic relaxation or deceleration (characterized by reduced Krebs cycle flux and lower overall reducing activity). This decrease in basal metabolic rate compromises neither the mitochondrial membrane potential nor cellular viability.

Our results agree with the findings of Gao et al., who showed that inhibition of mitochondrial tricarboxylic acid cycle or electron transfer chain mitigated lipid peroxide accumulation and ferroptosis [48]. We consider that our findings are consistent with a decrease of mitochondrial oxidation in the presence of zileuton. Our observations could account for a subtle modulation of mitochondrial activity in the presence of the 5-LOX inhibitor that might not exert deleterious effects on cellular physiology, given that cellular viability was not impaired by the compound. Altogether, our results support the protective action of the 5-LOX inhibitor against oxidative stress and ferroptosis.

Summarizing, zileuton may induce a cytoprotective state characterized by Nrf2-mediated antioxidant priming and mitochondrial metabolic deceleration without compromising cellular viability or bioenergetic integrity. Treatment with zileuton might promote a mild, non-cytotoxic oxidative stimulus (indicated by subtle increases in TBARS and protein oxidation) that triggers Nrf2 phosphorylation and the subsequent upregulation of key antioxidant enzymes. This preconditioned antioxidant network blunts H_2_O_2_-induced protein oxidation, rescues the depleted GSH/GSSG ratio, and confers protection against ferroptosis. Concurrently, zileuton may induce a state of metabolic quiescence marked by citrate accumulation and decreased succinate and malate levels, signaling a reduced flux through the Krebs cycle. This slowing of metabolic turnover would reduce the generation of reducing equivalents (explaining the observed decrease in AlamarBlue reduction) without disturbing ψm. Overall, these findings suggest that zileuton could coordinate Nrf2-dependent antioxidant defenses and an adaptive metabolic slowdown to shield cells from severe pro-oxidant insults and ferroptotic cell death. 

## Supplementary Information

Below is the link to the electronic supplementary material.

Supplementary Material 1

## Data Availability

Data available from the corresponding author upon reasonable request.

## Abbreviations

AA: arachidonic acid
CAT: catalase
CM-H_2_DCFDA: 5-(and-6)-chloromethyl-2’,7’-dichlorodihydrofluorescein diacetate, acetyl ester
FCCP: carbonyl cyanide-4-(trifluoromethoxy)phenylhydrazone
GClc: catalytic subunit of glutamate-cysteine ligase
GSSG: oxidized glutathione
GSH: reduced glutathione
HBSS: Hank’s balanced salts
Ho-1: heme oxygenase-1
H_2_O_2_: hydrogen peroxide
5-LOX: 5-lipoxygenase
Ψm: mitochondrial membrane potential
Nqo1: NAD(P)H quinone oxidoreductase 1
Nrf2: nuclear factor erythroid 2-related factor
PLA2: phospholipase A2
PSCs: pancreatic stellate cells
ROS: reactive oxygen species
RT-qPCR: quantitative reverse transcription-polymerase chain reaction
SOD: superoxide dismutase
Tps: thapsigargin
TMRM: tetramethylrhodamine methyl ester perchlorate
Zil: zileuton

## Declarations

### Generative Ai and Ai-assisted technologies in the manuscript preparation process

Neither generative AI nor AI-assisted technologies were used in the writing of this manuscript
