# Supplementary material for: Zileuton protects pancreatic stellate cells from oxidative stress and ferroptosis by modulating the Nrf2 pathway and mitochondrial activity
Source: J Physiol Biochem. 2026 Sep 16;82(1):93. doi: 10.1007/s13105-026-01224-9 (PMC13582277; doi:10.1007/s13105-026-01224-9)
Supplement: Supplementary file 1 [file 13105_2026_1224_MOESM1_ESM.pdf]

FIGURE 4_R1

p-Nrf2

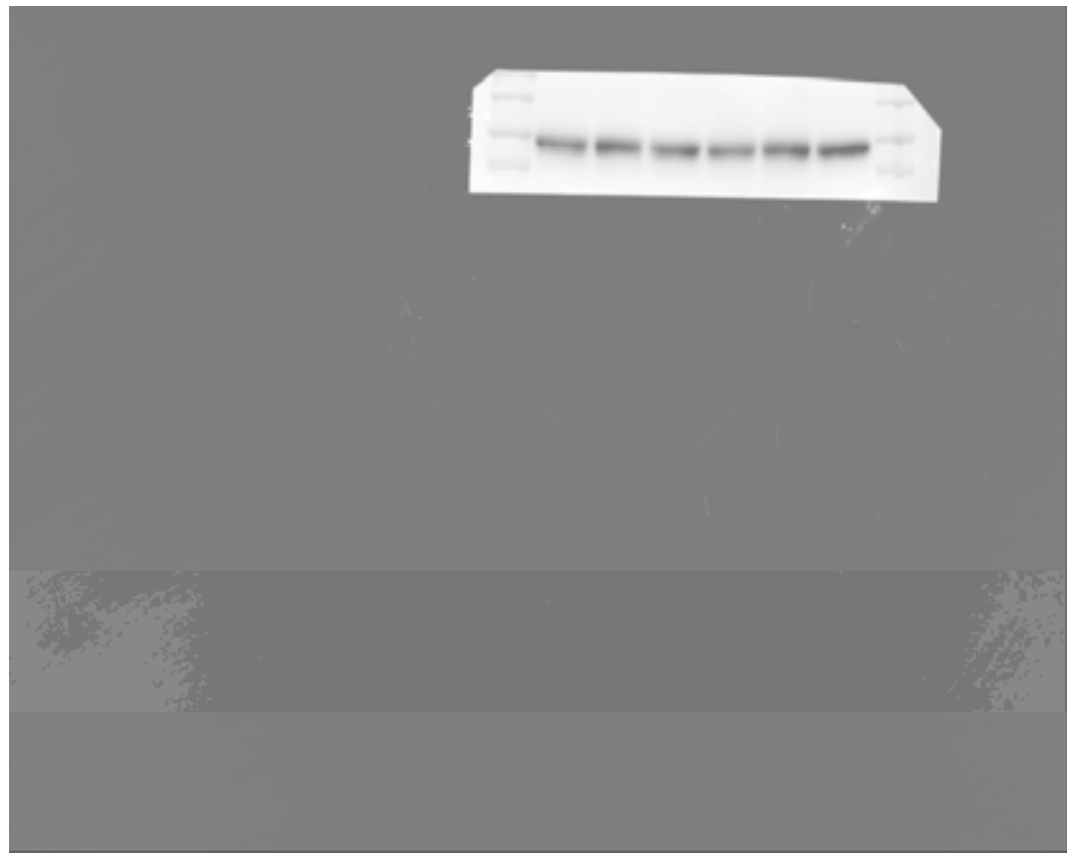

β-actin

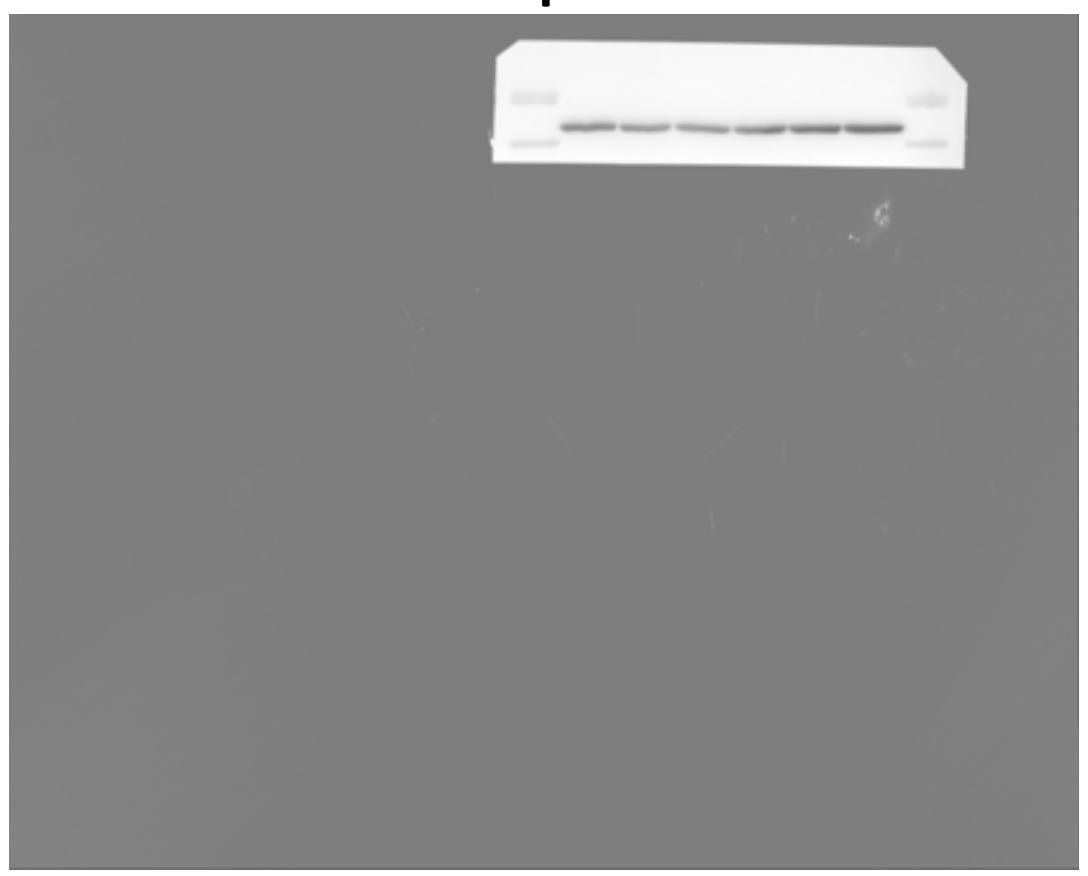

Nrf2

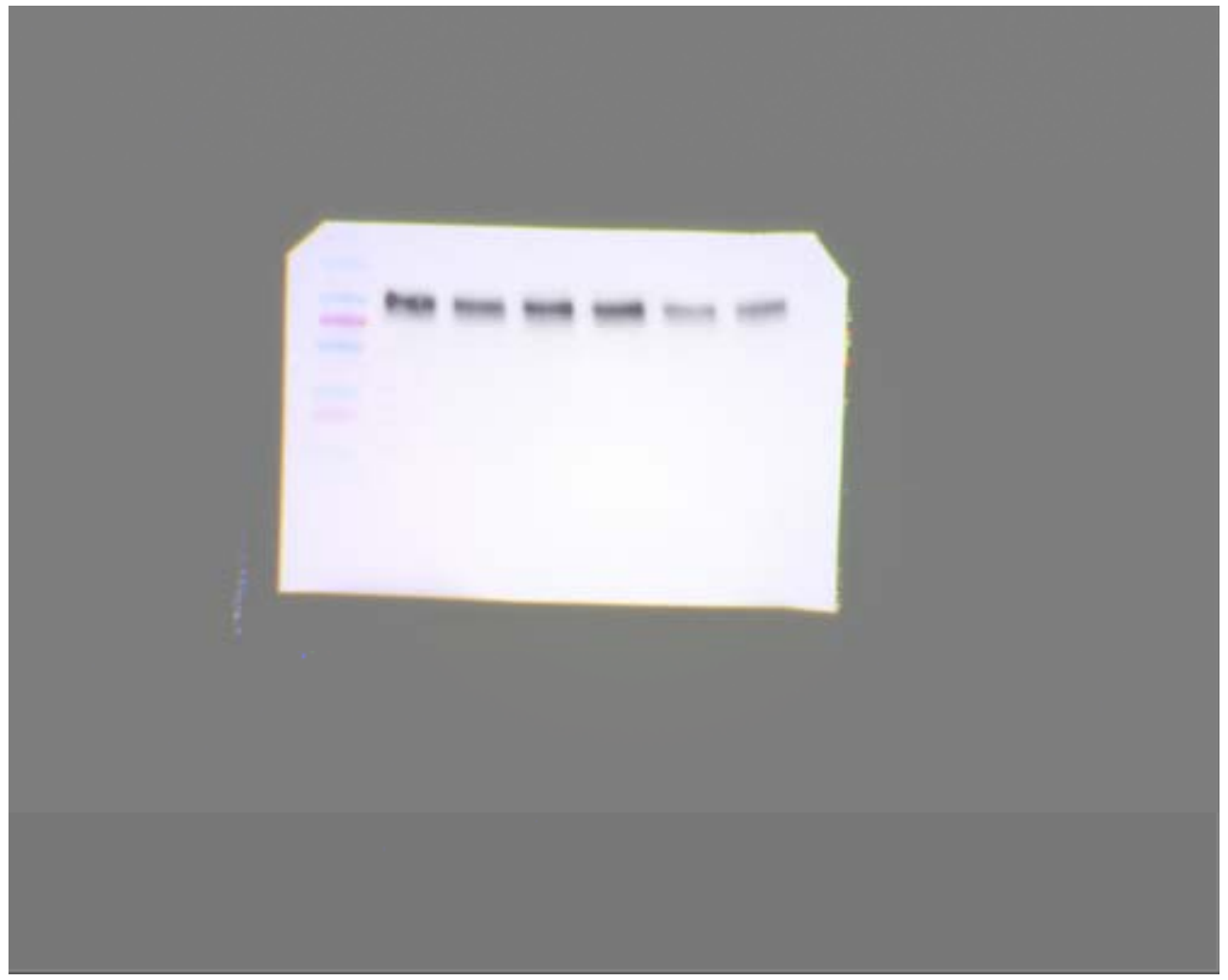

Lane 1: non-treated cells
Lane 2: Zileuton 10 μM

FIGURE 5_R1

A

SOD-1

B

SOD-2

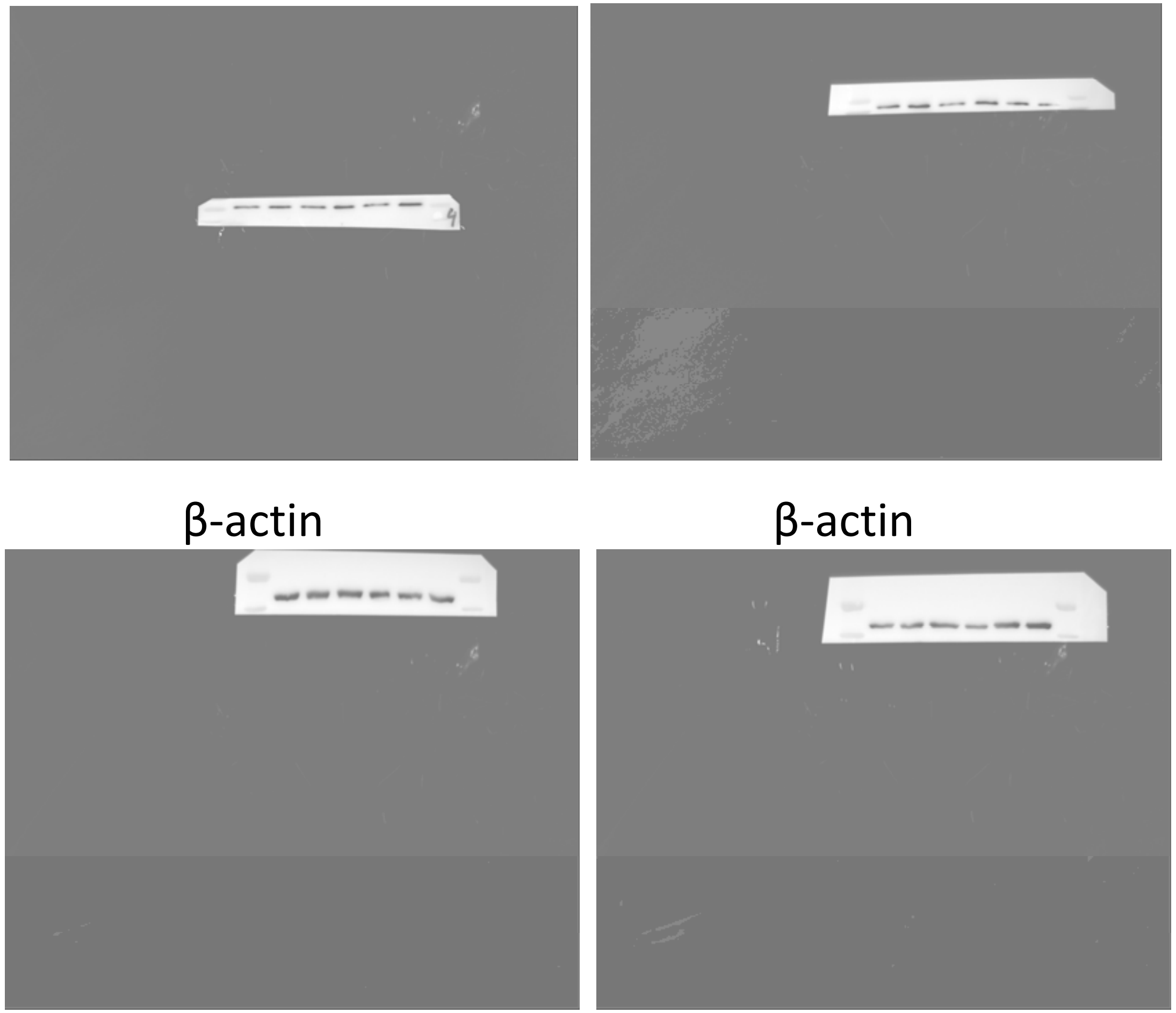

β-actin

β-actin

Lane 1: non-treated cells
Lane 2: Zileuton 10 μM
